# A case report of comprehensive treatment for primary intraspinal carcinosarcoma

**DOI:** 10.3389/fonc.2024.1479193

**Published:** 2025-01-07

**Authors:** Chang Yan, Chao-Jun Zhang, Jun-bao Wei, Hui-wen Liang, Song Qu

**Affiliations:** ^1^ Department of Radiation Oncology, Guangxi Medical University Cancer Hospital, Nanning, Guangxi, China; ^2^ Department of Pathology, Guangxi Medical University Cancer Hospital, Nanning, Guangxi, China

**Keywords:** carcinosarcoma, central nervous system tumors, radiotherapy, chemotherapy, comprehensive treatment, rare disease

## Abstract

**Background:**

Carcinosarcoma is a rare and highly aggressive biphasic malignant tumor. To date, no cases of primary intraspinal carcinosarcoma have been reported.

**Case presentation:**

This study reports a case of a 36-year-old female with primary intra dural extramedullary carcinosarcoma. The patient underwent surgery at initial diagnosis, followed by two courses of radiotherapy due to residual tumor, as part of a comprehensive antitumor treatment. Despite good tumor control, she ultimately died of respiratory failure.

**Discussion:**

This is the first reported case of primary intra dural extramedullary carcinosarcoma, detailing its imaging characteristics, pathological morphology, and treatment process. The tumor was responsive to radiotherapy. The rapid progression of intraspinal carcinosarcoma suggests it may be underdiagnosed or underreported, highlighting the need for more cases for clinical evaluation and treatment strategies.

## 1 Introduction

The simultaneous presence of both carcinomatous and sarcomatous components within a single tumor is a rare occurrence, and such tumors are typically referred to as carcinosarcomas, which are highly invasive. It is essential to distinguish carcinosarcoma from sarcomatoid carcinoma, despite their similar nomenclature. Sarcomatoid carcinoma is a type of carcinoma that originates from epithelial tissue but exhibits sarcoma-like structural features under the microscope (1). The exact pathogenesis of carcinosarcoma remains unclear.

Carcinosarcoma is commonly found in the female reproductive system, including the uterus and ovaries (2, 3). The currently accepted treatment involves surgical resection followed by postoperative chemotherapy, but the prognosis remains poor (4). Carcinosarcoma can also occur in other rare locations, including the skin (5), bladder (6), lungs (7), salivary glands (8), thyroid (9), pancreas (10), gums (11), and liver (12). The gastrointestinal tract can also be affected by carcinosarcoma, with cases observed in the esophagus, stomach, colon, and rectum, where the esophagus is the most commonly involved site (13–16).

The occurrence of carcinosarcoma in the central nervous system is extremely rare, with most cases previously reported as metastases. We conducted a comprehensive literature review and identified 9 cases of intracranial metastasis (17–25), 2 cases of intraspinal metastasis (26, 27), and 2 cases involving both intracranial and intraspinal involvement (28). Additionally, one article reported a case of carcinosarcoma in the pineal gland of a 9-year-old male child, where the authors, based on pathological findings, suggested that the tumor’s origin in some malignant mixed tumors of the pineal gland might be from local neural elements rather than peripheral ones (29).

This paper reports a case of carcinosarcoma originating in the spinal canal, with the carcinoma component being poorly differentiated keratinizing squamous carcinoma and the sarcoma component being undifferentiated sarcoma. A review of the literature reveals that primary intraspinal carcinosarcoma has not been previously reported, and its biological behavior and clinicalpathological features have not been documented. Therefore, we present this case to share its clinical and pathological characteristics and treatment process.

## 2 Case presentation

**Figure 1:**
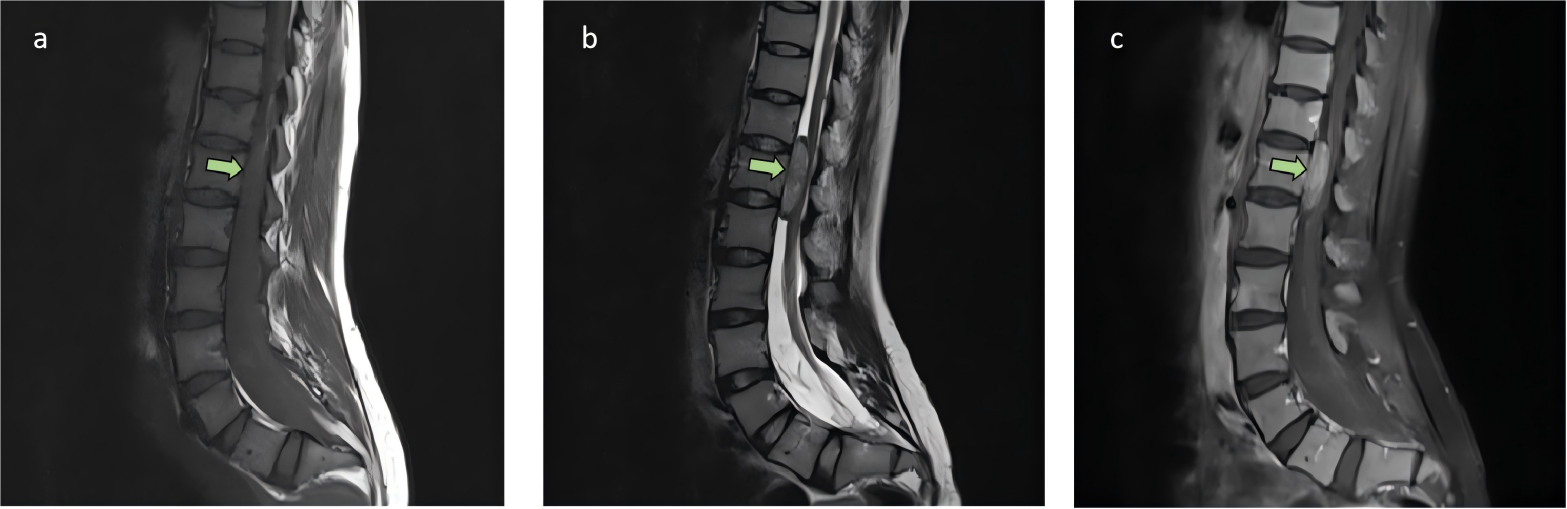
The sagittal MRI images from the patient’s initial consultation. A space-occupying lesion was observed within the intra dural extramedullary space from the 11th thoracic vertebra to the 1st lumbar vertebra, with clear borders and compression at the spinal cord level of the 12th thoracic vertebra, as indicated by the arrow. The lesion appears isointense on T1-weighted imaging **(A)**, while on T2-weighted imaging **(B)**, it predominantly shows slightly higher intensity, with punctate high signals within. There is marked enhancement observed on the contrast-enhanced scan **(C)**. No significant abnormal signals were observed elsewhere in the spinal canal.

**Figure 2:**
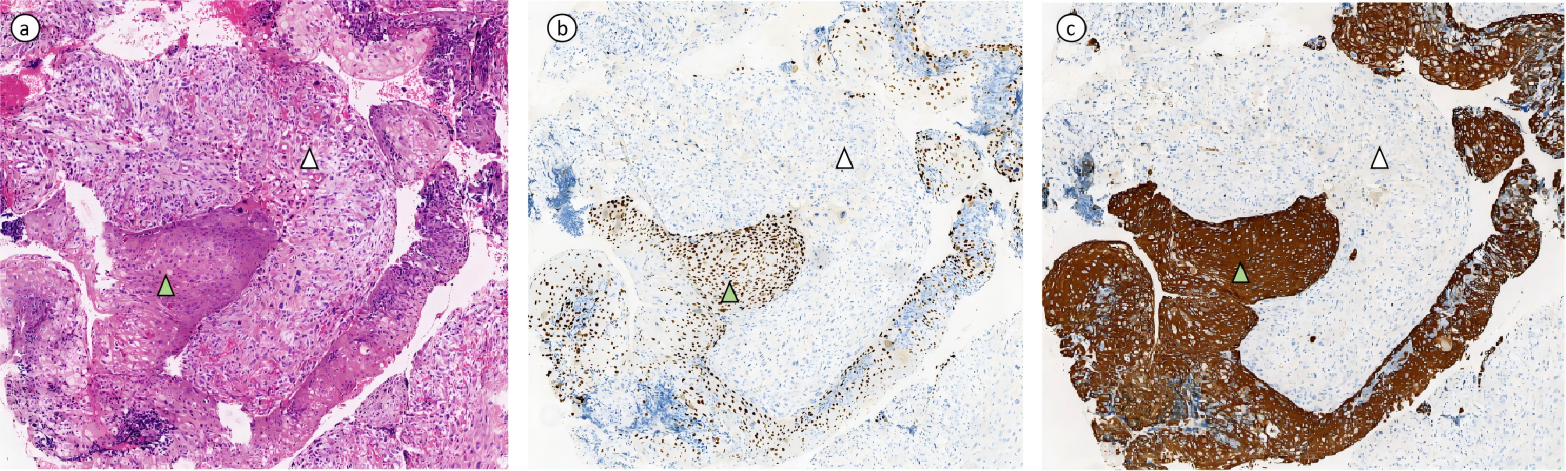
(Intraspinal, Thoracic 11 - Lumbar 1) Sarcomatoid Carcinoma. **(A)** Hematoxylin and eosin (HE) stained section showing tumor composed of epithelial and mesenchymal components. The carcinoma component is a poorly differentiated squamous cell carcinoma with keratinization, marked with green triangle symbols in the image. The sarcomatoid component is an undifferentiated sarcoma, exhibiting spindle-shaped and epithelioid morphology with pleomorphism, indicated by white arrowheads in the image. **(B, C)** depict immunohistochemical stained sections. The carcinoma component shows positive expression of P40 and CK5/6, while the mesenchymal component is negative for P40 and CK5/6.

A 36-year-old female presented to a local hospital with “sudden onset of paraplegia, sensory abnormalities, and urinary and fecal incontinence for three days.” Magnetic resonance imaging(MRI) indicated a space-occupying lesion in the intra dural extramedullary space from T11 to L1, with clear borders and compression at the spinal cord level of the 12th thoracic vertebra. The lesion appears isointense on T1-weighted imaging (**Figure 1A**), while on T2-weighted imaging (**Figure 1B**), it predominantly shows slightly higher intensity, with punctate high signals within. There is marked enhancement observed on the contrast-enhanced scan (**Figure 1C**). After ruling out contraindications, the patient underwent posterior laminectomy and decompression at the T11-L1 level, as well as tumor resection within the spinal canal. After performing a two-thirds laminectomy at each segment, an adjuvant durotomy was carried out. The tumor was located intradurally and extramedullary, appearing grayish-white to grayish-brown with dark red necrotic areas. It was irregularly shaped, solid, and relatively hard, with severe compression and adhesion of the dural sac nerves. Using a sharp knife, the middle portion of the dural sac was incised, and the tumor was carefully dissected and removed from the nerves using a nerve dissector. There were no intramedullary components, and no intraoperative complications occurred. Postoperative pathology revealed intraspinal carcinosarcoma from T11 to T12 (**Figure 2**), containing both epithelial and mesenchymal components.

**Figure 3:**
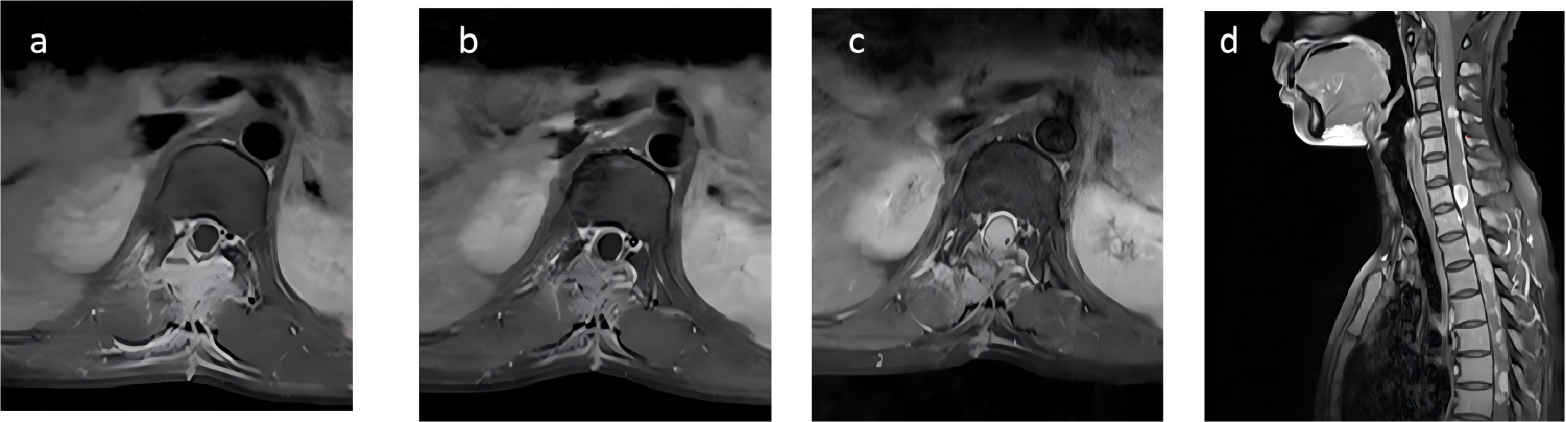
Initial MRI scan of the patient upon first examination at our hospital, showing patchy signal changes in the Th11-12 surgical area and adjacent soft tissues, with significant enhancement on contrast-enhanced scans **(A)**. Following the first round of radiotherapy, the signal changes have slightly decreased compared to before, with reduced enhancement on subsequent scans **(B)**. Two months later, MRI indicated an increase in the nodular lesion at T12, with invasion into the spinal cord **(C)**. The sagittal view showed the appearance of multiple new small nodules indicating intramedullary dissemination within the spinal cord **(D)**.

Postoperatively, her symptoms did not significantly improve, prompting her to seek further treatment at our hospital. Physical examination revealed decreased deep and superficial sensations below the groin on the right side, decreased deep sensation but preserved superficial sensation on the left side; muscle strength of iliopsoas and quadriceps was grade 3 bilaterally, ankle dorsiflexion on the left was grade 2-, and 0 on the right; increased muscle tone in the right lower limb; normal muscle strength and tone in both upper limbs; physiological reflexes were present, and no pathological reflexes were elicited. Laboratory tests, including complete blood count, liver and kidney function, and coagulation profile, were within normal ranges. MRI indicated postoperative changes at T11-12, with soft tissue enhancement in the surgical area suggestive of residual tumor (**Figure 3A**). Positron Emission Tomography-Computed Tomography(PET/CT) showed increased metabolic activity in the surgical area with no abnormal tumor metabolism elsewhere, ruling out the possibility of a primary tumor at another site. Surgical re-evaluation found no indication for further surgery.

**Figure 4:**
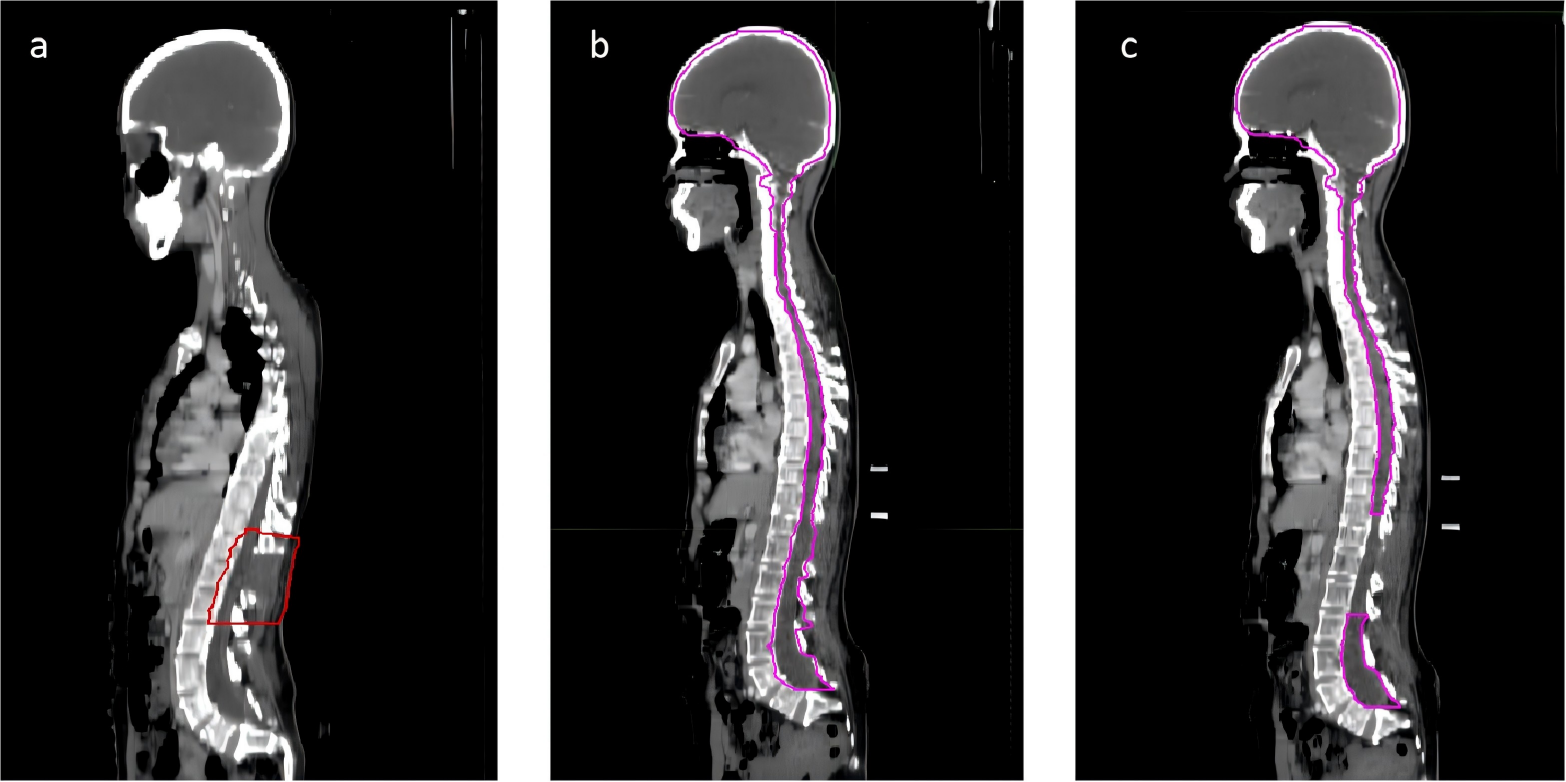
The sagittal view of the radiation therapy target areas for the patient. The target area for the patient’s first round of radiation therapy is defined as the region where tumor remnants are visible on imaging **(A)**. During the initial 10 sessions of the second round of radiation therapy, the target area encompasses the entire central nervous system **(B)**. In the subsequent 10 sessions of the second round of radiation therapy, the target area excludes the Gross Tumor Volume (GTV) delineated during the first round of therapy and is limited to the brain and some segments of the spinal cord **(C)**.

Considering the metabolic activity observed in the surgical area and the aggressive, recurrent nature of carcinosarcoma, radiotherapy was deemed necessary. She underwent tomotherapy in our department, with GTV defined as the visible residual tumor and surgical area, extended by 5 mm to form PCTV. The prescribed dose was PGTV 44.0 Gy in 22fractions (**Figure 4A**). During radiotherapy, she was given dexamethasone and mannitol to reduce intracranial pressure. Post-radiotherapy, she reported slight improvement in left lower limb strength but remained unable to move freely. Follow-up MRI, conducted 10 days later, revealed a reduction in soft tissue enhancement in the surgical area, suggesting effective radiotherapy (**Figure 3B**).

A multidisciplinary team(MDT) recommended palliative chemotherapy to control tumor progression, recognizing the limited benefit in improving lower limb strength, sensory abnormalities, and urinary and fecal functions. Considering the patient’s weak constitution (ECOG score 3), the GT regimen, with relatively lower toxicity, was suggested. She received the first cycle of GT regimen palliative chemotherapy with gemcitabine 1g/m² (1.6g) on days 1 and 8, and docetaxel 75mg/m² (120mg) on day 1. She developed grade III leukopenia and urinary tract infection, leading to the cancellation of gemcitabine on day 8.

Two months later, the patient developed further muscle weakness and numbness in both upper limbs. MRI showed an increase in the nodular lesion at T12, with invasion into the spinal cord (**Figure 3C**). The sagittal view showed the appearance of multiple new small nodules indicating intramedullary dissemination within the spinal canal (**Figure 3D**). Due to the poor response to chemotherapy, the patient returned to department of radiation oncology for further care. Typically, the recommended radiotherapy dose should not exceed 45Gy to avoid radiation-induced spinal cord injury. However, considering the patient’s good response to radiotherapy and the difficulty in restoring lower limb function, and considering the excellent conformality of the previous tomotherapy, the patient and her family were informed about the situation and agreed to a second course of intensity modulated radiation therapy, mainly to extend survival. She underwent the second course of palliative radiotherapy, with CTV defined as the entire central nervous system for the first 10 sessions (**Figure 4B**). For the subsequent 10 sessions, CTV excluded the GTV from the first course, focusing on the brain and part of the spinal cord (**Figure 4C**). PCTV was expanded by 5mm, with a dose of PCTV 40Gy in 20 fractions. During radiotherapy, the patient experienced increased numbness and tremors in both upper limbs, as well as increased muscle tone in both lower limbs. These symptoms were partially alleviated after treatment with valproate and dexamethasone.

During radiotherapy, the patient received two cycles of toripalimab 240mg for immunotherapy. Subsequently, she was continued maintenance therapy with anlotinib 12mg daily (days 1-14 of a 21-day cycle) for targeted treatment. Due to personal reasons, MRI follow-up was not performed. Post-radiotherapy CT indicated a reduction in the nodular lesion at T12, making it difficult to measure size accurately, and multiple small nodules in the spinal canal appeared less distinct or reduced in number, suggesting a positive treatment response. She continued maintenance therapy with anlotinib, achieving stable tumor control. However, three months later, the patient died of respiratory failure after her family discontinued treatment due to difficulties with phlegm excretion at home. Her overall survival was 11 months.

## 3 Discussion

Carcinosarcoma, also referred to as malignant mixed Müllerian tumors (MMMTs), is characterized by the presence of both carcinoma and sarcoma components. While it most commonly occurs in the uterus, carcinosarcoma can also be found in other sites such as the lungs, esophagus, ovaries, and gallbladder (30–33). Carcinosarcoma has a poor prognosis, and the primary treatment involves surgical resection, with radiotherapy and chemotherapy used to improve outcomes further (3, 34, 35).

Although carcinosarcoma is rare, its incidence has been steadily increasing over the past few decades, possibly due to advancements in cancer screening, imaging, and pathological techniques (36). To date, no cases of primary intraspinal carcinosarcoma have been reported. Currently, only 2 cases of carcinosarcoma occurring within the spinal canal as metastatic tumors have been reported (27). Our comprehensive analysis of the patient’s PET/CT and CT scans revealed no abnormal masses in other parts of the body, ruling out metastasis in this case. Initially, the patient presented with paraplegia and sensory abnormalities in both lower limbs, as well as urinary and fecal incontinence, primarily due to the space-occupying effect within the spinal canal.

The etiology of carcinosarcoma remains unclear. Several hypotheses have been proposed regarding its pathogenesis: 1. Transformation Hypothesis (3): Suggests that the sarcomatous component is derived from the carcinomatous component through epithelial-to-mesenchymal transition (EMT). 2. Collision Hypothesis (37): This hypothesis suggests that carcinosarcoma arises from the differentiation of two distinct cell types, namely mesenchymal cells and epithelial cells. 3. Stem Cell Hypothesis (38): Posits that carcinosarcoma originates from pluripotent stem cells, differentiating into both epithelial and mesenchymal lineages during proliferation. In this case, the most likely mechanism is differentiation from pluripotent stem cells. Genetic testing may help determine the origin of carcinosarcoma.

In this case, CT and MRI features of intraspinal carcinosarcoma resembled those of other extramedullary intradural and extradural tumors. On imaging, the tumor was isointense on T1WI, slightly hyperintense on T2WI, and showing marked uniform enhancement on contrast scans. This can lead to misdiagnosis as meningioma, schwannoma, or chordoma. Due to the heterogeneous mixture of carcinosarcoma components, cytological smears often fail to capture all elements, suggesting that fine-needle aspiration biopsy is of limited value for diagnosing intraspinal carcinosarcoma. The best method may be the gross pathological result after tumor resection, with immunohistochemical and electron microscopy results providing additional diagnostic support.

While the incidence of carcinosarcoma has been increasing, it remains relatively low overall. Some studies suggest that combination therapy may improve survival rates, but prospective clinical trials to support this are challenging to conduct (2, 39, 40). There is still a lack of guidelines for the diagnosis and treatment of carcinosarcoma, and standardized treatment protocols are currently unavailable (3, 41). Surgical resection is a widely accepted therapeutic option for spinal canal tumors, even though recurrence may occur in the follow-up period (42, 43). In the present case, surgery was initially performed; however, recurrence was observed postoperatively. Considering the highly malignant nature and significant recurrence rate of carcinosarcoma, we opted for postoperative radiotherapy and salvage radiotherapy for this case. After two courses of radiotherapy, the tumor was well controlled. Postoperative radiotherapy for patients with uterine carcinosarcoma can significantly improve survival rates, especially for those with stage I-II disease over the age of 70 (44). Additionally, small-scale studies on ovarian carcinosarcoma suggest that radiotherapy should be considered in certain cases to benefit patients (45). After radiotherapy and MDT discussion, we decided on palliative chemotherapy using the GT regimen. The two chemotherapy drugs in this regimen have synergistic effects and are commonly used in treating soft tissue sarcomas. They have demonstrated efficacy in some refractory or recurrent cases and demonstrating significant antitumor activity in metastatic soft tissue sarcoma patients, with manageable side effects (46). Some studies and clinical trials have also evaluated the effectiveness of the GT regimen in uterine carcinosarcoma patients. Although the sample sizes are limited, results indicate that this chemotherapy regimen is effective in some patients (47). However, this case demonstrated poor tolerance to the GT regimen, as the eighth day of the first chemotherapy cycle not being completed. Subsequent anti-tumor treatment was switched to immunotherapy with toripalimab and targeted therapy with anlotinib. Although research on the application of toripalimab in carcinosarcoma is relatively limited, other PD-1/PD-L1 inhibitors have demonstrated potential efficacy in studies on certain types of sarcomas. Research indicates that pembrolizumab and nivolumab have some efficacy in certain soft tissue sarcomas, and their combined use can provide synergistic effects. On one hand, targeted therapy can inhibit tumor angiogenesis, improve the tumor microenvironment, and enhance the effectiveness of immunotherapy (48); on the other hand, immunotherapy can enhance immune surveillance, further inhibiting tumor growth. For primary spinal canal carcinosarcoma patients who cannot tolerate GT regimen chemotherapy, switching to maintenance therapy with toripalimab and anlotinib is a reasonable and potentially promising alternative. Although specific clinical data are limited, based on the mechanisms of immunotherapy and targeted therapy and their application experiences in other tumors, this combination treatment provides effective antitumor activity and improves patient prognosis.

## 4 Conclusion

We report the first case of a rare primary carcinosarcoma in the spinal canal. The initial symptoms were neurological sensory and motor dysfunctions caused by a space-occupying lesion in the spinal canal, which were confirmed by imaging and postoperative pathological examination. The patient initially underwent surgery, followed by recurrence postoperatively. Subsequently, the patient received radiotherapy and exhibited good tolerance and a positive response. This case provides valuable insights into the clinical diagnosis and treatment of carcinosarcoma in the spinal canal, and offers new perspectives for research in related fields, particularly concerning the effects of radiotherapy and prognosis.

## Data Availability

The original contributions presented in the study are included in the article/supplementary material. Further inquiries can be directed to the corresponding author.
